# Seasonal and spatial distribution of ixodid tick species feeding on naturally infested dogs from Eastern Austria and the influence of acaricides/repellents on these parameters

**DOI:** 10.1186/1756-3305-6-76

**Published:** 2013-03-19

**Authors:** Georg G Duscher, Andrea Feiler, Michael Leschnik, Anja Joachim

**Affiliations:** 1 Institute of Parasitology, Department of Pathobiology, University of Veterinary Medicine Vienna, Veterinaerplatz 1, Vienna A-1210, Austria; 2 Small Animal Clinic, Department for Companion Animals, University of Veterinary Medicine Vienna, Veterinaerplatz 1, Vienna, A-1210, Austria

**Keywords:** *Ixodes ricinus*, *Dermacentor reticulatus*, Biting locations, Seasonality, Permethrin, Fipronil

## Abstract

**Background:**

Effective control of tick infestation and pathogen transmission requires profound knowledge of tick biology in view of their vector function. The particular time of the year when the different tick species start to quest and the favoured sites on the canine host are of major interest. The efficacy of acaricides/repellents to control ticks in the field requires observation.

**Methods:**

To address these issues, 90 dogs, grouped in “untreated”, “acaricide/repellent” (permethrin) and “acaricide only” (fipronil) animals and subjected to tick infestation under natural conditions in Burgenland (Eastern Austria), were examined. The number and species of ticks occurring during and outside the protection time was evaluated during a period of 11 months and the biting location on the dogs’ skin was recorded.

**Results:**

Of the 700 ticks collected, the most common species in that particular walking area was *Ixodes ricinus*, followed by *Dermacentor reticulatus* and *Haemaphysalis concinna*. Regarding the on-host activity, *D. reticulatus* displayed more infestations in early spring and late autumn, whereas *I. ricinus* occurred almost one month later in spring and one month earlier in autumn. *H. concinna* followed a monophasic pattern of activity with a peak in summer. The preferred feeding sites of the ticks on the dogs were on the head, neck, shoulder and chest. This distribution over the dog’s body was not influenced by the use of the drugs, although on the whole fewer ticks (22.5% of all ticks) were found during the protection time. Interestingly, differences occurred with the use of drugs compared to non-protected dogs with regard to the infestation over the year. Acaricide-treated dogs displayed a higher prevalence in April, May and September, whereas dogs of the acaricide/repellent group showed a higher infestation in March, July, October and November.

**Conclusion:**

The different tick species display different on-dog activity peaks over the year, during which particular canine diseases can be expected and predicted, considering the specific incubation times for each pathogen.

The tick species occurring in this study do not seem to choose particular sites on the dogs. Their arrival place seems to represent the attachment and consequently the feeding sites. The use of acaricides leads to a significantly (p<0.01) lower number of infesting ticks but no change of the distribution pattern on the dogs was observed.

## Background

Ixodid ticks of various species occur frequently in central Europe. The most common species described are *Ixodes ricinus, Ixodes canisuga, Ixodes hexagonus, Dermacentor reticulatus,* and *Haemaphysalis concinna*[1]. However, the species vary with regard to frequency, host specificity, seasonal activity and ability to transmit pathogens. Dogs are frequently infested with *I. ricinus*, which can transmit *Anaplasma phagocytophilum*, *Borrelia burgdorferi* sensu latu, *Rickettsia helvetica* and tick-borne encephalitis (TBE) virus [2,3]. They can also be infested with *D. reticulatus*, the vector for *Babesia canis canis*[2], which is frequently found in Hungary [4] and is assumed to be endemic in the Eastern parts of Austria along the border with Hungary. Furthermore, this tick species acts as a vector for TBE, *Francisella tularensis, Rickettsia conorii*[5] and *Rickettsia slovaca*[6]. *H. concinna*, rarely found on dogs [4], can presumably carry TBE, *Francisella tularensis*[5], *Rickettsia sibirica*[2] and *Coxiella burnettii*[7]. Other tick species are occasionally found feeding on dogs but there is only scarce information about their role as vectors of canine pathogens, e.g. *I. hexagonus* for borreliae [8] and *Theileria annae*[9]. In this study we investigated (i) the seasonal occurrence of the different tick species on dogs, (ii) the spatial distribution pattern of ticks on the dogs and (iii) the effect of acaricides on the number of infesting ticks and on the temporal and spatial distribution of ticks on dogs.

### The seasonal distribution of ticks throughout the year

To date, very little is known about the mechanism and development of allochrony of different tick species occurring in the same area. Studies in Hungary on dogs [4], foxes [10] and questing ticks [11] describe different activity peaks of several tick species. The activity pattern of each tick species is of great importance with regard to the differing vector capacity and the control measures to be taken. Yet no information about the tick species occurring on dogs is available for Austria. Due to the proximity to Hungary and the similarities in landscape and climate, the distribution of tick species and their activity patterns on dogs originating from Eastern Austria (Burgenland) was assumed to resemble that of the neighbouring country.

### The attachment sites of ticks on dogs

There is only scanty information regarding the distribution of ticks on the dog showing the preferred infestation sites, so-called predilection sites, which might contribute to control measures such as mechanical removal. The most common assumption regarding the behaviour of ticks looking for a place to feed is that they wander around searching for thin and soft skin in sheltered places on the host after being wiped off the vegetation. However, Földvári and Farkas [4] found most ticks on dogs on the head, followed by the neck and legs. These places represent the first arrival places for questing ticks on the animal and are also less prone to grooming.

### The effect of acaricides/repellents on the occurrence and distribution of ticks on the canine host

Repellent and killing effects, especially for permethrin, cannot readily be differentiated. This acaricide is repellent in low doses and killing in high doses with no particular cut-off. Both effects result in fewer or dead ticks, representing the overall effect of this acaricide. This can be compared to the purely acaricidal effect of fipronil. According to the manufacturers’ descriptions of commercial spot-on acaricides/repellents, a high rate of killed and/or repelled ticks can be expected. Therefore the number of ticks on untreated dogs or dogs not (re-) treated within the given protection time should be much higher than that on protected dogs. In contrast, treatment is not expected to have an influence on the seasonality of ticks, i.e. although fewer ticks can be expected on treated dogs, the proportion at various times of the year should be the same as on untreated animals. Furthermore, no shift of the feeding ocation on treated dogs to places further away from the acaricide application sites (neck) was expected. According to Lüssenhop et al. [12] the effect of acaricides is distributed homogeneously on the dog’s skin, indicating that there is no reduction of the concentration of the active component with increasing distance from the application site.

The aim of the study was to describe the seasonal dynamics of natural tick infestations of dogs, to identify areas on dogs to which ticks might be more attracted and to investigate whether the application of a topical acaricide influences these parameters.

## Methods

### Animals

Ninety clinically healthy adult dogs (58 females, 32 males, aged 6 months to 13 years) of different breeds recruited from a small animal clinic in Burgenland, Eastern Austria, an area endemic for the ticks mentioned, were enrolled in this study. Animals [13] were allocated to three groups of equal numbers as follows: the group “permethrin” was treated with a commercially available spot-on acaricide/repellent (permethrin, Exspot®, Intervet GmbH, Austria), the group “fipronil” was treated with an acaricide (fipronil, Frontline®spot on, Merial, France); the “untreated” group received no treatment. In the untreated group, 2 dogs died before the end of the study in winter 2008/2009: the causes of death were not related to ticks or to tick-transmitted pathogens. Furthermore, in each of the treated groups one dog was excluded due to poor owner compliance. Tick removal of the four dogs was completed by December 2008 and the animals were subsequently excluded from the serological investigations. Application time and intervals of the acaricides were subject to the owner’s decision. Consequently, the treated animals were grouped further according to dogs within protection time (i.e., the recommended treatment interval for the two ectoparasiticides) and outside the protection times. The time spans were added up and delivered the total protective vs. non-protective times. Owners were asked to record the treatment and to immediately report on symptoms like fever, anorexia, lameness, or neurological signs that may have occurred in their dogs during the year.

### Sampling

For an examination period of eleven months (February – December 2008), the dogs were walked daily in an area known to be endemic for ticks and which is defined by the four coordinates N48°6^′^54"E16°42^′^9‵; N48°17^′^56"E16°49^′^49"; N47°55^′^2"E16°36^′^50‵; N47°51^′^51‵E16°50^′^56‵ and covers about 192 km^2^. January was left out because of the expected low tick abundance and the reduced walking ability of the owners. The owners examined their dogs for ticks immediately afterwards. Attached ticks were removed by the owner, stored at −20°C and subsequently classified for species, sex and stage [5].

The exact site of each tick found attached on the dog was recorded on a pre-drawn schematic picture. This locations were transferred into “arcmap 9.3.1" (Synergis Wien ESRI®, 2009), enabling calculation of kernel dot density. Other calculations were performed in Excel® 2002 (Microsoft, Washington) and SPSS v. 17 (SPSS Inc., Chicago, Illinois).

The experiments were approved by the institutional ethics committee (University of Veterinary Medicine Vienna) and the Austrian Ministry for Science and Research (GZ68.205/25-II/10b/2010).

## Results

### Temporal distribution of tick species and stages

Within 11 months a total of 700 ticks (684 feeding ticks and 16 copulating males) were collected and classified according to species and stage.

**Figure 1:**
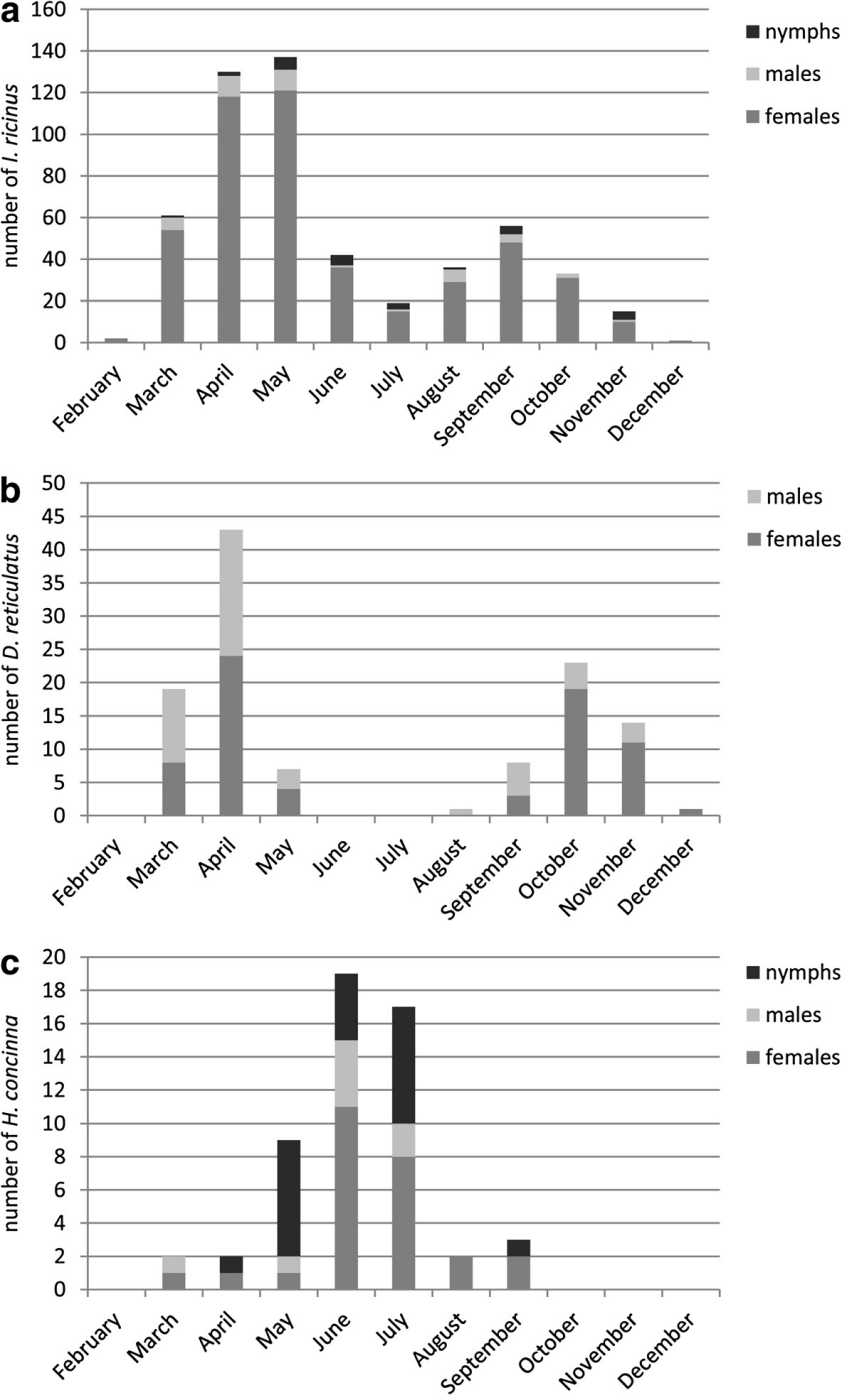
**Distribution of tick species (a: *Ixodes ricinus*, b: *Dermacentor. reticulatus*, c: *Haemaphysalis concinna*) and stages over the year 2008.**

*I. ricinus* (n= 532, including the 16 copulating males) was the most common tick species found (Figure 1a), followed by *D. reticulatus* (n=107; Figure 1b), and *H. concinna* (n=54; Figure 1c). Three *I. hexagonus* (one female, two nymphs) were found in March and April and four *I. canisuga* (one female and three nymphs) were discovered over the year. With regard to stages, of the 532 *I. ricinus* 4.9% were nymphs, 7.7% males and 87.4% females. Of 107 *D. reticulatus*, 34.6% were males and 65.4% were females, while the 54 *H. concinna* specimens comprised 37.0% nymphs, 14.8% males and 48.2% females (Figure 1). For further analysis only attached ticks were used. Copulating males were not included as they were not found feeding on the host. Therefore the overall number of ticks analysed was 684.

*I. ricinus* and *D. reticulatus* were detected in all months with peaks in spring and autumn, while *H. concinna* occurred mostly in summer (Figure 1).

### Attachment sites of ticks on dogs

**Figure 2:**
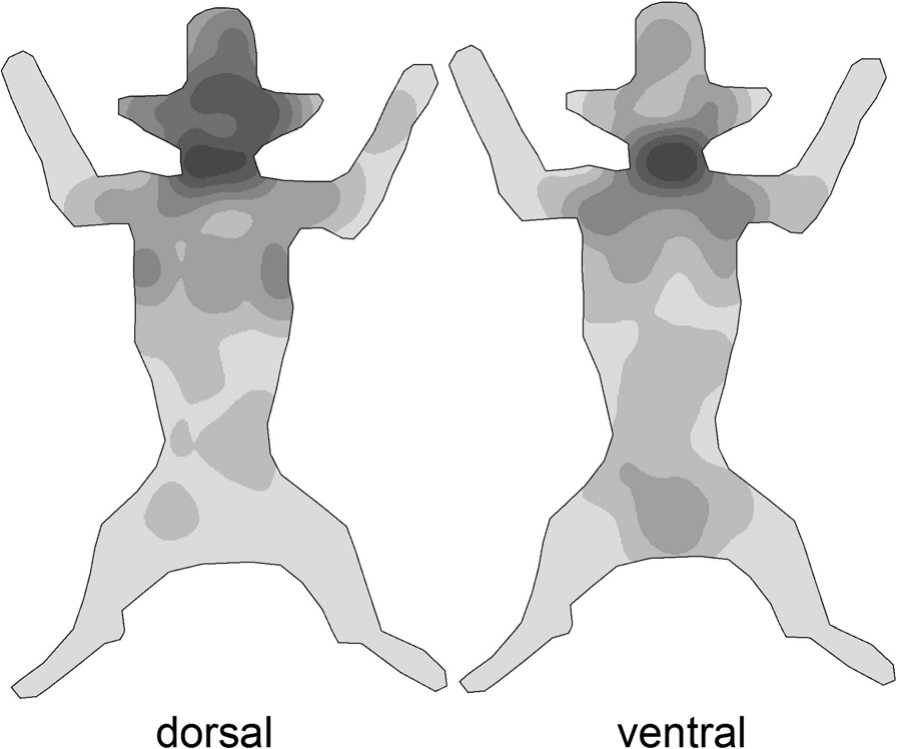
**Density of cumulative tick infestation (n=90 dogs), left: dorsal view, right: ventral view.** A darker grey in the figure indicates a higher density of ticks.

The body surface was divided into seven different areas. Most ticks were removed from the head and ears, chest and neck regions of the infested dogs (Figure 2).

### Effect of acaricides

**Figure 3:**
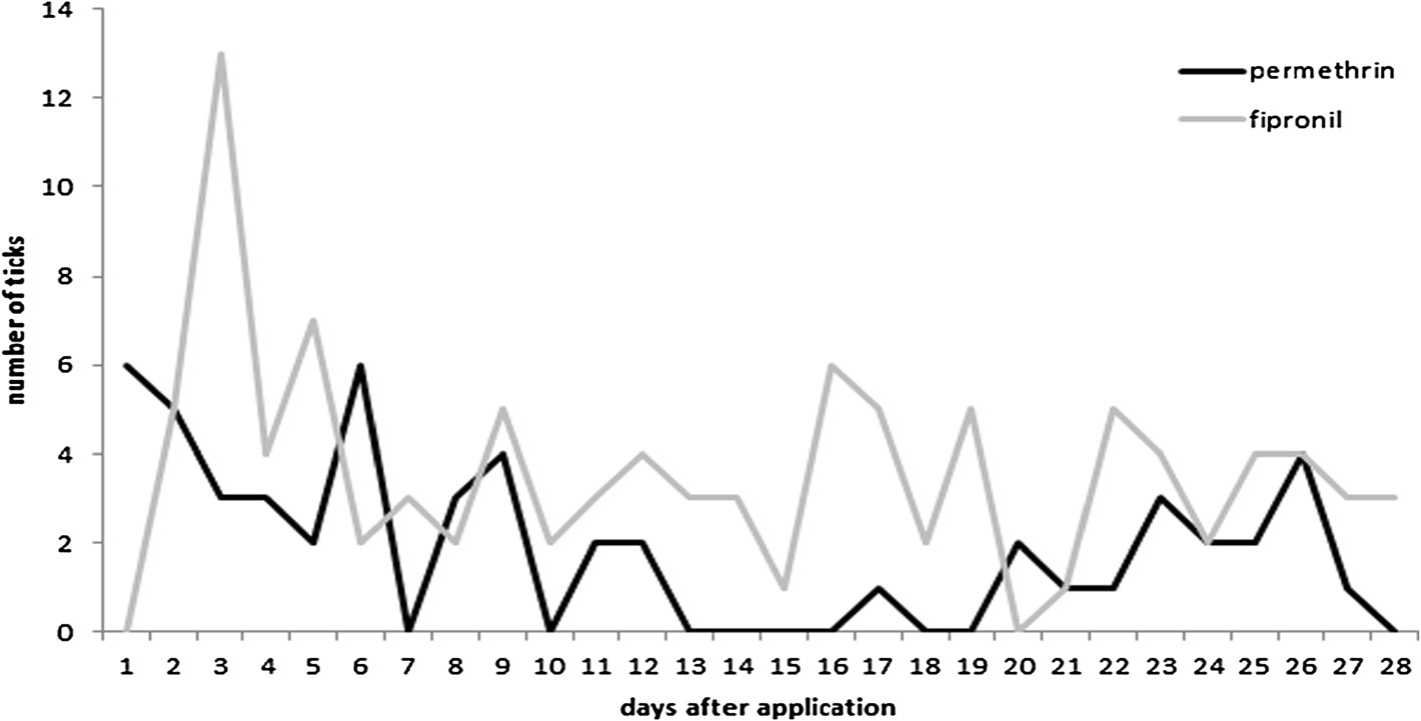
Number of ticks removed from the dogs after treatment with permethrin (n=53, black line) or fipronil (n=101, grey line) in relation to the number of days after application.

Of the 684 feeding ticks 77.5% were found during the non-protected time (including the total number found on the untreated dogs), whereas 22.5% were removed during the time the dogs were calculated to be protected (Table 1). 7.7% of all ticks were found in the acaricide/repellent group and 14.8% in the acaricide group. There was no clear trend in the efficacy of either permethrin or fipronil with regard to reduction of tick infestation related to time after application (Figure 3). There was no significant difference in the species composition in non-protected vs. protected animals (p=0.298) or between non-treated, acaricide-treated and acaricide/repellent-treated dogs (p=0.187).

**Table 1:** Number of days and ticks collected from the animals in the non-protected (untreated), acaricide/repellent (permethrin) and acaricide only (fipronil) groups

	**Days**	**Ticks**	**Ticks/day**	**Ticks/month**
Untreated (n=30)	24990	530	0.021	0.636
Permethrin (n=30)	2856	53	0.019	0.557
Fipronil (n=30)	2548	101	0.040	1.189
**Total**	**30394**	**684**		

The number of dogs per group is given in brackets (n). If ticks were removed from dogs of the acaricide or acaricide/repellent groups outside the protection time, the dogs were considered to be “untreated” and the time was added to the number of days of the untreated group.

### Influence of acaricide treatment on seasonality

**Figure 4:**
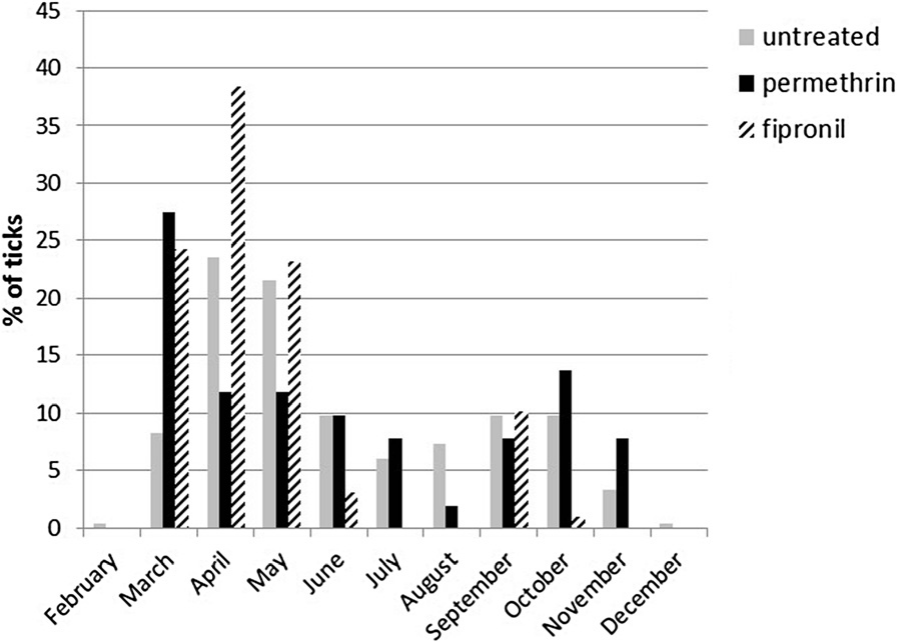
Percentage of tick species in non-protected (untreated), acaricide/repellent (permethrin) and acaricide only (fipronil) groups collected monthly over the year 2008.

Untreated dogs, those in the permethrin group and those in the fipronil group showed infestations with 0.64, 0.56 and 1.19 ticks per month (Table 1), respectively. The seasonal occurrence differed significantly between the groups (Chi^2^ test, p<0.01; Figure 4). Permethrin-treated dogs displayed a higher rate of tick infestation than the non-protected and the fipronil groups in March, July, October and November. The fipronil group had higher tick infestation rates in April, May and September.

During the period they were expected to be protected, dogs treated with permethrin or fipronil had significantly (Chi^2^ test; p<0.01) fewer nymphs (0.7%) than the unprotected dogs (9.2%) (Table 2).

**Table 2:** Proportion of instars of all ticks (n=684) in the untreated (non-protected) and treated (protected: permethrin + fipronil) groups

	**% of untreated dogs (n=30)**	**% of treated dogs (n=60)**
Nymphs	9.2*	0.7*
Males	10.1	12.0
Females	80.7	87.3

(* indicates a significant difference between the groups, p<0.01).

### Influence of acaricides on the attachment sites of ticks on dogs

The Kruskal-Wallis test revealed no significant differences (p=0.55) between non-protected, acaricide/repellent and acaricide only groups with regard to the attachment site of ticks on the dogs (Table 3).

**Table 3:** Number of ticks found on the different regions of the dog in the untreated, permethrin- and fipronil-treated groups

	**Untreated (%)**	**Permethrin (%)**	**Frontline (%)**	**Total (100%)**
Head	174 (80.9)	8 (3.7)	33 (15.3)	215
Neck	121 (76.6)	18 (11.4)	19 (12.0)	158
Chest	85 (73.9)	11 (9.6)	19 (16.5)	115
Back	27 (75.0)	3 (8.3)	6 (16.7)	36
Belly	31 (77.5)	5 (12.5)	4 (10.0)	40
Pelvic region	22 (84.6)	1 (5.3)	3 (16.0)	26
Legs	74 (78.7)	5 (5.3)	15 (16.0)	94
**Total**	**534 (78.1)**	**51 (7.5)**	**99 (14.5)**	**684**

## Discussion

### Temporal distribution of tick species and stages

The season for the start of tick activity is of major interest, especially for veterinarians and pet owners, to determine the best practice for countermeasures against tick infestation. The most common ixodid species, *I. ricinus* and *D. reticulatus*, were detected throughout the investigation period, but showed different activity patterns. Allochrony of different species is supposed to be based on diverging ecological mechanisms of reproductive isolation in the course of evolution [11]. Especially for sympatric species with similar host ranges, the mechanism is assumed to be an important driving force for separation during evolution [11].

The major tick species found on the dogs was *I. ricinus*, in contrast to the findings 2005 in Hungary, where *D. reticulatus* was most frequent [4]. Two years later, during a larger scale study involving 1424 ticks, *I. ricinus* was found to be most frequent on dogs from Hungary [14], which is consistent with our results. The activity of *I. ricinus* was demonstrated during numerous studies especially in relation to TBE outbreaks, and is described as a double-peak activity over the year in central Europe [3]. The major peak in *I. ricinus* activity occurs in spring, with a smaller one in autumn. This pattern could be confirmed in the present study, which found peaks in April/May and September in accordance with studies of questing ticks in Hungary in the same year [11]. In previous investigations in Hungary, the same activity period of *I. ricinus* ticks on foxes was observed [10]. A different pattern was observed in the tick infestation of foxes in Thuringia [15], where *I. ricinus* occurred monophasically from April to September. *I. ricinus* collected from dogs [4] in Hungary showed a biphasic activity pattern with the start of the highest infestation levels varying slightly from March/April and September/October, possibly due to climate differences. The variations might be regarded as natural fluctuations over the years; e.g. a sudden increase in temperature in early spring is assumed to trigger the start of the tick questing season much more efficiently than it would during an overall mild winter [11]. Similarly, a rapid temperature decrease in the fall stopped the activity of more ticks than it would during a mild fall [16]. The full effects of climate and the different bioclimatic areas on tick behaviour are still a matter of investigation [16].

Interestingly, *D. reticulatus* also showed a biphasic cycle but occurred one month earlier (March) in spring and one month later (October) in autumn than *I. ricinus.* This tick seems to be able to quest under colder environmental conditions [17]. The activity of the species is of great importance in dogs, as *D. reticulatus* is responsible for transmitting canine babesiosis, which is very often lethal. Hornok [11] observed a similar two-peak pattern of questing ticks but with a higher number of ticks during the autumn peak. On foxes [10] the effect was even more obvious. The latter study did not differentiate between the stages, possibly leading to a higher number of ticks found due to the endophilic lifestyle of *D. reticulatus* larvae and nymphs [18], where these instars are adapted to their hosts and remain in sheltered places such as burrows, nests or hollows [18]. Therefore foxes, which also spend much time of their live in sheltered dens, are more frequently infested by these instars and display a slightly different infestation pattern than that of dogs. On Hungarian dogs, *D. reticulatus* ticks were found during the same time period (March/April and September/October) as *I. ricinus*[4]. In contrast to this previous study, no earlier occurrence of *D. reticulatus* was observed, possibly because in the Hungarian study ticks were collected from different climatic regions throughout Hungary, whereas in the present study the sampling area was restricted to the eastern parts of Austria. On foxes from Thuringia, *D. reticulatus* displayed a low abundance with a restriction to the east and north [15], so no temporal analysis is available.

In contrast to the two species mentioned before, *H. concinna* followed a monophasic activity pattern. This tick normally feeds on wild deer and is only rarely found on dogs [5]. Very little is known about the species’ biology and life cycle. Activity peaks have been described in March-June and in October [5]. In the present study *H. concinna* occurred in a single peak in June/July, indicating a preference for higher temperatures. Similar results were obtained for questing *H. concinna* in Hungary with an activity peak in May/June [11], and for ticks on dogs between April and July [4]. Széll and co-workers [10] observed the same pattern on foxes. Different weather conditions at the sampling sites could be the explanation for earlier activity of *H. concinna* in Hungary. There is also evidence from previous studies that in Hungary the activity for *H. concinna* can be one month later [10]. Again, there may be natural fluctuations in the start of the season influenced by climatic conditions in different regions and years, although the seasonal distributions follow the principal activity pattern (monophasic or biphasic).

### Attachment sites of ticks on dogs

It is a common belief that ticks, after reaching the host by being wiped off the vegetation, wander around to seek a suitable location to take their meal. The feeding place is assumed to be sheltered, with soft and thin skin. If the exploration phase is not successful, the mouthparts can be withdrawn and feeding resumed elsewhere [19]. In this study the majority of ticks was located on more exposed (head, shoulder, chest) body regions and also on those with thicker skin (shoulder, chest). This is in concordance with previous investigations in Hungary [4], which generally found the same infestation sites on dogs. The position of the feeding locations of the ticks led us to the assumption that these body parts correspond to the arrival points of the questing ticks on the dog, when the dog is moving through grass with its head on the ground. Experimental studies are needed to confirm this conclusion. The head, shoulder and chest are at the front while walking through the vegetation so these areas are the places with the most frequent tick contact and fewer “predilection sites” of the ticks per se. Ticks crawling on the fur are surely under higher risk of being wiped off again while the dog continues to move through the vegetation. Inside the coat, ticks can move only slowly. Unlike fleas, which are laterally flattened and therefore can easily crawl through dense hair, ticks are dorso-ventrally flattened. This body shape enables them to crawl on the surface of the fur but represents a handicap to moving through dense coats. In contrast, in humans, where hair is scarce, ticks tend to search for more sheltered areas: they are distributed further away from the site of placement during artificial infestation of humans [20]. During natural infestation [21] ticks are found on the upper and lower extremities in equal proportions, followed by the abdomen.

### Effect of acaricides

The number of ticks was generally lower after the use of permethrin or fipronil, although 7.7% and 14.8%, respectively, of all ticks were still removed during the protection times. The number of ticks found on dogs treated with fipronil was almost twice as high as in the non-protected group. The reason could be the higher exposition pressure in this group, although as all groups used the same area for walking this explanation is vague. The efficacy of either acaricide did not show any differences during the protection time. Ticks were removed during the entire period, even immediately after application. It is important to note that no information about tick viability could be obtained in this study. Hence the ticks, although attached, might have already been killed by the acaricide. Nevertheless, the protective effect was not as high as expected even in the permethrin group. One possibility is that a higher proportion of repelled ticks than others wander around, enabling the dog owners to find them crawling in the fur more easily. The use of ectoparasiticides in the field is known to deliver lower efficacy than in the laboratory. Possible reasons could be lack of owner compliance or misuse, e.g. too little substance reaching the skin, bathing or swimming immediately after treatment, and the selection for acaricide-resistant ticks as observed recently for *Rhipicephalus microplus*[22]. Interestingly, there was a significant difference between the non-protected and the permethrin and fipronil groups with regard to the seasonality. Ticks are strongly influenced by temperature and host seeking behaviour increases with temperature. In the case of the *R. sanguineus,* higher temperatures have been described to induce a higher level of aggression, leading to the infestation of host species other than the primary ones [23]. It is also possible that the repellent effect is suppressed with increasing temperature but this will have to be investigated in separate studies.

There was an obvious effect of acaricides/repellents on nymphs. These stages are known to be more susceptible to ectoparasitic treatment [24].

The location of the feeding site of ticks on the dog did not differ between protected and non-protected dogs. The assumption that tick biting behaviour is not influenced with increasing distance from the application site [12] could thus be confirmed.

## Conclusions

Although ticks can be found in almost all seasons, the different species cluster periodically over the year, starting with *D. reticulatus*, followed by *I. ricinus*, then *H. concinna* and again *I. ricinus* and ending with *D. reticulatus*. Tick-borne infections related to certain tick species can be expected during these periods after the respective incubation times.

In dogs it seems that the feeding site is close to the arrival site and the ticks waste neither energy nor time in seeking more suitable places. This conclusion awaits confirmation in laboratory trials. Therefore the feeding sites of ticks on dogs do not seem to represent an innate preference of the ticks.

Although fewer ticks were found on treated dogs, their distribution pattern was largely the same of that of the untreated group. Distribution was independent of the distance between the application site of the spot-on and the biting location of the tick. The feeding location seems to be much more restricted to the arrival location than previously thought. This finding must be investigated more precisely to elucidate the feeding behaviour of ticks on their hosts.

## Competing interests

The authors declare no competing interests.

## Authors’ contributions

GD analysed the data and wrote the manuscript. AF participated in its design and performed the coordination. ML participated in the design of the study and statistical analysis. AJ participated in the analysis and helped to draft the manuscript. All authors read and approved the final manuscript.
